# Determinants of age at menarche in the UK: analyses from the Breakthrough Generations Study

**DOI:** 10.1038/sj.bjc.6605978

**Published:** 2010-11-02

**Authors:** D H Morris, M E Jones, M J Schoemaker, A Ashworth, A J Swerdlow

**Affiliations:** 1 Section of Epidemiology, Institute of Cancer Research, Sir Richard Doll Building, 15 Cotswold Road, Sutton, Surrey SM2 5NG, UK; 2 Breakthrough Breast Cancer Research Centre, Institute of Cancer Research, London SW3 6JB, UK

**Keywords:** diet, epidemiology, exercise, growth, menarche, puberty

## Abstract

**Background:**

Early menarche increases breast cancer risk but, aside from weight, information on its determinants is limited.

**Methods:**

Age at menarche data were collected retrospectively by questionnaire from 81 606 women aged 16–98, resident in the UK and participating in the Breakthrough Generations Study.

**Results:**

Menarche occurred earlier in women who had a low birthweight (*P*_trend_<0.001), were singletons (*P*<0.001), had prenatal exposure to pre-eclampsia (*P*<0.001) or maternal smoking (*P*=0.01), were not breastfed (*P*_trend_=0.03), were non-white (*P*<0.001), were heavy (*P*_trend_<0.001) or tall (*P*_trend_<0.001) compared with their peers at age 7 and exercised little as a child (*P*_trend_<0.001). Menarcheal age increased with number of siblings (*P*<0.001) independently of birth order, and had an inverse association with birth order after adjustment for sibship size (*P*<0.001). In a multivariate model, birthweight, ethnicity, weight, height, exercise, sibship size and birth order remained significant, and maternal age at birth became significant (positive association, *P*<0.001).

**Conclusion:**

Age at menarche was influenced by both pre- and post-natal factors, and these factors may affect breast cancer risk through this route.

Early menarche increases risk of breast cancer, probably as a consequence of earlier onset of ovulation and cyclic exposure to female sex hormones, and hence potentially an increased cumulative exposure to these hormones over the course of life (Colditz *et al*, 2006). It is widely accepted that the substantial variation in age at menarche between individuals is due to both genetic and environmental factors (Towne *et al*, 2005), with the strongest associations shown for childhood weight and body mass index (Moisan *et al*, 1990; Cooper *et al*, 1996; Koprowski *et al*, 1999; Freedman *et al*, 2002). Specifically, heavier girls reach menarche much earlier than lighter girls, and it has been postulated that body fat is the component of weight that causes this relationship (Frisch, 1987). Taller girls (Moisan *et al*, 1990; dos Santos Silva *et al*, 2002; Freedman *et al*, 2002) and those who do not exercise regularly (Frisch, 1987) also have earlier menarche, but it is uncertain whether these factors function independently of weight.

It is unclear which other factors are important. In particular, the effects of prenatal and early life factors on age at menarche remain uncertain, in the main due to a lack of adequately powered studies. For example, there is weak evidence that menarche is delayed in girls who were heavier at birth (Romundstad *et al*, 2003; Opdahl *et al*, 2008) and who were breastfed during infancy (Novotny *et al*, 2003; Ong *et al*, 2009), but contradictory results exist for both of these findings (Moisan *et al*, 1990; Adair, 2001; Windham *et al*, 2004; Blell *et al*, 2008).

We investigated factors that might affect menarcheal age in 81 606 women participating in the Breakthrough Generations Study (BGS), to improve understanding of the causation of menarche both as a significant biological event and as a breast cancer risk factor.

## Materials and methods

The BGS is a cohort study of breast cancer aetiology, for which all women aged 16 or older who live in the UK are eligible. The South Thames Multicentre Research Ethics Committee approved the study. Recruitment began in 2003 and 111 595 women have joined. The initial participants were supporters of the Breakthrough Breast Cancer charity, and women who referred themselves. Participants nominated other women and the majority of participants were recruited in this way. Therefore, there are a large number of BGS participants who have other family members in the study.

Participants were eligible for the analyses if they reported their age at menarche on the baseline questionnaire (*N*=100 594). We excluded 6409 women with a history of breast cancer or ductal carcinoma *in situ* at study entry (who were greatly over-represented), 16 women whose menarche occurred later than age 20 years, because often their menses were initiated by exogenous hormone treatment and 2 women who reported intermittent menses at ages 3 or 4 years followed by a later menarche. If two or more first-degree relatives were eligible for the analysis, then only the youngest relative was included (*N*_excluded_=12 561). These analyses therefore included 81 606 women.

Most variables were reported on a detailed baseline questionnaire that was completed by all participants. The exceptions were the breastfeeding and maternal smoking variables, which were obtained by record linkage to the mother's questionnaire, and so these analyses were restricted to women whose mothers were also study members (*N*=9278). Furthermore, data on maternal smoking were only available if the subject was the first-born child (*N*=6340).

Age at menarche was reported in whole years with an option for participants who had never menstruated. For childhood weight, participants were asked ‘At the age of 7 years, were you thinner, about the same or heavier than other girls of your age?’. An analogous question was asked for height at age 7. Birthweight was reported in either grams or pounds and ounces. Siblings were only included in the sibship size and birth order analyses if they shared a mother with the subject, as paternal half-siblings could not have influenced the subject's prenatal environment and were less likely to have shared the subject's childhood home.

### Statistical analysis

Univariate linear regression was applied to assess differences in age at menarche between different levels of the exposure variable. A multivariate model was chosen using backward selection and terms that were not significant at the 5% level were removed. This model implies that the effects of variables are additive. Model selection was performed using the subset of participants who provided information for every variable under consideration for the stepwise model but the final estimates included the larger set of participants who provided information for every variable in the final model. Breastfeeding and maternal smoking were not included in the multivariate model due to the large amount of missing data for these variables (*N*=72 328 and 75 266 respectively). Analyses were repeated excluding participants who were aged 30 years or older at study entry, because recall of age at menarche and its determinants might be less accurate in the older women (Towne *et al*, 2005). These results are not shown unless they differed materially from the unrestricted analyses. Analyses were performed in Stata 10.0 (StataCorp, 2007) and all *P*-values were two sided.

## Results

Baseline characteristics of the 81 606 women included in these analyses are shown in Table 1. Mean age at menarche was 12.7 years (standard deviation=1.5 years, range=7–20 years). Menarche occurred before age 11 in 4.8% of participants, at ages 11–14 in 85.1% and at age 15 or older in 10.1%. In univariate analyses (Table 2), menarche occurred earlier in women who had a low birthweight (*P*_trend_<0.001), whose mother had pre-eclampsia or eclampsia (*P*<0.001), or who smoked (*P*=0.01) during the pregnancy with the subject, who were not breastfed (*P*_trend_=0.03), were not white (*P*<0.001), were heavier (*P*_trend_<0.001) or taller (*P*_trend_<0.001) than their peers at age 7 years, did not exercise (*P*_trend_<0.001), had no siblings (*P*_trend_<0.001) and were the first-born child (*P*_trend_<0.001). Twins had a later menarche than singletons (*P*<0.001; data not shown) but, when analysed by type of twin, the difference was significant for participants who were part of an opposite-sex dizygotic or a monozygotic twin pair but not those who were part of a same-sex dizygotic twin pair (Table 2). There was no association with maternal age at birth (*P*_trend_=0.15) or being a vegetarian before age 8 years (*P*=0.18). These results were all similar after adjustment for socio-economic status and birth year (data not shown).

In the multivariate model (Table 2), the associations with birthweight (*P*_trend_<0.001), ethnicity (*P*<0.001), weight (*P*_trend_<0.001), height (*P*_trend_<0.001), exercise (*P*_trend_<0.01) and number of siblings (*P*_trend_<0.001) remained significant. A higher birth order (*P*_trend_<0.001) and a younger maternal age at birth (*P*_trend_<0.001) were now associated with an earlier menarche. Twinning and exposure to pre-eclampsia or eclampsia had a significant effect in univariate analyses, but not in the multivariate model.

The multivariate model suggests that the variables can combine to give large differences. For example, a non-white girl who was much heavier and taller than her peers at age 7 could reach menarche nearly 2 years earlier on average than a white girl of average weight and height, assuming that all other factors were the same (difference=−22.8 months, 95% confidence interval=−25.0 to −20.7 months).

In subgroup analyses, birthweight had a positive association with menarcheal age within each childhood weight category (*P*_trend_<0.001; Table 3) and in first- and second-born women (first: effect=0.4, *P*_trend_<0.001, *N*=28 033; second: effect=0.3, *P*_trend_=0.02, *N*=16 875), but not in women of a higher birth order (third: effect=0.1, *P*_trend_ 0.74, *N*=5822; fourth: effect=0.2, *P*_trend_=0.46, *N*=1646; fifth or higher=−0.5, *P*_trend_=0.31, *N*=740). In each height category, weight had a significant inverse association with menarcheal age (*P*_trend_<0.001; Table 3), and similarly within each weight category, height had an inverse association (*P*_trend_<0.001).

In the univariate analyses restricted to participants who were younger than age 30 years at recruitment, pre-eclampsia exposure did not have a significant effect (*P*=0.08) and the association with exercise was stronger than in the unrestricted analyses (effect=2.8 months).

In additional analyses (data not shown), the effect of birthweight remained significant after adjustment for gestation length (*P*_trend_<0.001, *N*=31 484), as did the effect of twin status after adjustment for birthweight (*P*<0.01, *N*=56 049). When analysed separately, age at menarche increased by 1.1 (*P*_trend_<0.001) and 0.8 (*P*_trend_<0.001) months per sister and brother respectively. There was a significant negative association with birth order after adjustment for sibship size only (*P*_trend_<0.001).

## Discussion

In this study of more than 80 000 women, age at menarche was influenced by both pre- and post-natal factors, and some of these factors appear to interact with each other.

Menarche was delayed in girls who were heavier at birth, which agrees with weak evidence from some previous studies (Cooper *et al*, 1996; Romundstad *et al*, 2003; Opdahl *et al*, 2008), although many differing findings exist (Adair, 2001; Windham *et al*, 2004; Tam *et al*, 2006). This association appears at first to be contradictory because a high birthweight is associated with a higher childhood weight (Danielzik *et al*, 2004), which was a risk factor for early menarche in our study and many others (Moisan *et al*, 1990; Cooper *et al*, 1996; Koprowski *et al*, 1999; Freedman *et al*, 2002). However, within each childhood weight category, birthweight had a positive association with age at menarche. This indicates that girls who had more catch-up growth during their early years had an earlier menarche, as suggested previously (dos Santos Silva *et al*, 2002). The positive association with birthweight was only present in first- and second-born women, who are likely to be smaller at birth than women with a higher birth order (Selvin and Janerich, 1971).

Previous findings for twins are inconsistent (Moisan *et al*, 1990; Kaprio *et al*, 1995; Anderson *et al*, 2007), but we found that twins had a later menarche than singletons. The differences between twins and singletons were not a consequence of differences in birthweight because the associations strengthened after adjustment for this factor.

Our study adds to the growing evidence that women with prenatal exposure to cigarette smoke have an earlier menarche. Of the three previous studies that have investigated this factor, two also found this result (Windham *et al*, 2004; Rubin *et al*, 2009), albeit non-significantly in the smaller of the two studies, whereas one found no association (Terry *et al*, 2009). We only had information on prenatal smoke exposure for first-born women whose mother was also a study member, but this should not bias the results because it seems very unlikely that selection into the study would differ by prenatal smoke exposure among this subset of participants.

It has been hypothesised, and there is evidence supporting this, that higher birthweight, maternal age and twinning probably increase breast cancer risk as they are associated with higher *in utero* oestrogen levels, whereas exposure to pre-eclampsia or eclampsia probably reduces breast cancer risk as they are associated with lower *in utero* oestrogen levels (Colditz *et al*, 2006). These are in the opposite direction to our findings for the relationships between these factors and age at menarche, suggesting that these prenatal factors affect age at menarche and breast cancer risk independently.

Other factors, however, were both associated with an earlier menarche in our results and are factors that lead to reduced risk of breast cancer. First, we found that girls who exercised more had a later menarche, which is consistent with previous findings (Frisch, 1987) and has been found to be associated with reduced risk of breast cancer (Colditz *et al*, 2006). Girls who exercise regularly are likely to be lighter, and though adjusting for childhood weight did not affect the result, increased exercise can change body composition by increasing muscle and reducing fat without an overall change in weight (Frisch, 1987). Second, in our study, women who were breastfed had a later menarche than those who were not, and there is evidence that being breastfed reduces breast cancer risk (Colditz *et al*, 2006). Several small studies have considered the relationship between age at menarche and breastfeeding, two of which found no association (Moisan *et al*, 1990; Blell *et al*, 2008) whereas two others accorded with our finding (Novotny *et al*, 2003; Ong *et al*, 2009). These latter two studies also found that subjects who had been formula-fed, as opposed to breastfed, had higher levels of body fat, which suggests one mechanism through which breastfeeding could delay menarche.

White women had a later menarche than non-white women in accord with fairly strong evidence from many (Koprowski *et al*, 1999; Freedman *et al*, 2002; Anderson *et al*, 2003; Windham *et al*, 2004), but not all (Bernstein *et al*, 1987), studies. The difference between the ethnic groups was strengthened after adjustment, and so might be a genetic effect.

We found that height was inversely associated with age at menarche, in accord with previous findings (Moisan *et al*, 1990; dos Santos Silva *et al*, 2002; Freedman *et al*, 2002). Only a few small studies adjusted for weight, and their findings were inconsistent (Cooper *et al*, 1996; Koprowski *et al*, 1999; Freedman *et al*, 2002). We found that the association with height was attenuated after adjustment for weight, but we used an imprecise measure of weight, hence the remaining association may have been due to residual confounding with weight.

We observed a significant positive association between number of siblings and age at menarche, which strengthened after adjustment for birth order. In previous, small studies, the relation with sibship size was unclear (Malina *et al*, 1997; Apraiz, 1999; Windham *et al*, 2004; Matchock and Susman, 2006). Our results suggest that childhood exposures or social effects related to family size could influence menarcheal age. Family size might be a correlate of socio-economic-related behaviours and environment but adjusting for socio-economic status did not affect the association with sibship size. Alternatively, it has been suggested that pheromonal cues from sisters might delay menarche (Matchock and Susman, 2006), but this argument would be more convincing if only number of sisters, and not brothers, was associated with the onset of menarche in our study, which was not the case.

After adjustment for sibship size, birth order had a significant inverse association with age at menarche. Previous studies found that birth order has a positive association with age at menarche (Malina *et al*, 1997; Apraiz, 1999; dos Santos Silva *et al*, 2002; Windham *et al*, 2004; Blell *et al*, 2008), but few studies have adjusted for sibship size and in those that did, which were much smaller than ours, no association with birth order was present after adjustment (Malina *et al*, 1997; Matchock and Susman, 2006). Birth order can be an indicator of prenatal effects, for instance first-born children are often lighter at birth than later children (Selvin and Janerich, 1971), however results were not affected by adjustment for birth weight.

Although association is far from indicating causality, causal relationships are plausible between age at menarche and several of these factors; for instance, weight loss can induce secondary amenorrhoea (Frisch, 1987). This may help to elucidate the mechanisms by which age at menarche affects breast cancer risk.

There are limitations to our study. The participants were volunteer recruits, and not a population-based random sample, which could potentially bias the associations. This might have affected the analyses for ethnicity because non-white participants were a more-selected, and perhaps differently selected, sample of all non-white women than the white participants. However, it is unlikely that there was material bias for other variables because, after adjusting for socio-economic status, women who had been breastfed, for example, were unlikely to have been selected on age at menarche in any different way from women not breastfed. As in many other large studies of this type, our data were retrospectively reported. Bean *et al* (1979) have shown that women can accurately recall age at menarche several decades after the event (range=17–53 years), with 90% of women accurately recalling their menarcheal age within 1 year. The retrospective reporting in this study may however be a particular problem when comparing women of different ages as recall errors for age at menarche and its determinants may be more likely for older women; the correlation between actual and recalled age at menarche is about 0.8 when reported up to 20 years after the event compared with 0.6 when a longer time has lapsed (Towne *et al*, 2005). Furthermore, the older women were a survivor population who might, in principle at least, be biased if combinations of age at menarche and its risk factors relate to early mortality. However, our results were generally similar when the analyses were restricted to the youngest women.

In conclusion, our results suggest that pre- and post-natal growth, body size and endogenous hormones affect age at menarche, and several of the causes of early menarche are themselves associated with increased breast cancer risk; thus, they may affect breast cancer risk through this route.

## Figures and Tables

**Table 1:** Descriptive variables for the 81 606 women included in the analysis

**Variables**	**Numbers and completeness of reporting**		**Description of variable**
**Continuous variables**	***N* (%) reporting**	***N* (%) missing**	**Median (range)**
Age at menarche, years	81 606 (100.0)	0 (0)	13 (7, 20)^a^
Age at entry, years	81 606 (100.0)	0 (0)	46 (16, 98)
Birthweight, g	56 678 (69.5)	24 928 (30.5)	3318 (734, 5958)
Maternal age at birth, years	77 336 (94.8)	4270 (5.2)	27 (12, 50)
Number of weeks breastfed^b^	9278 (11.4)	72 328 (88.6)	12 (0, 265)
Childhood exercise, h per week	77 796 (95.3)	3810 (4.7)	2 (0, 56)
Number of siblings	80 523 (98.7)	1083 (1.3)	1 (0, 13)
Birth order	75 541 (92.6)	6065 (7.4)	0 (0, 12)
			
**Categorical variables**	***N* (%) reporting**	***N* (%) missing**	***N* (%) in each category**
*Twinning*	80 567 (98.7)	1039 (1.3)	
Singleton			78 989 (98.0)
Same-sex dizygotic twin			526 (0.7)
Opposite-sex dizygotic twin			587 (0.7)
Monozygotic twin			465 (0.6)
			
*Pre-eclampsia or eclampsia* c	67 545 (82.8)	14 061 (17.2)	
Not exposed			64 375 (95.3)
Exposed			3170 (4.7)
			
*Maternal smoking* c,d	6340 (7.8)	75 266 (92.2)	
Not exposed			5710 (90.1)
Exposed			630 (9.9)
			
*Ethnicity*	81 438 (99.8)	168 (0.2)	
White			80 468 (98.8)
Mixed white/non-white			327 (0.4)
Non-white^e^			643 (0.8)
			
*Weight at age 7 years compared with peers*	80 335 (98.4)	1271 (0.2)	
Much thinner			3363 (4.2)
A little thinner			17 246 (21.5)
About the same			49 110 (61.1)
A little heavier			9492 (11.8)
Much heavier			1124 (1.4)
			
*Height at age 7 years compared with peers*	79 656 (97.6)	1950 (0.2)	
Much shorter			2431 (3.1)
A little shorter			13 725 (17.2)
About the same			41 444 (52.0)
A little taller			17 949 (22.5)
Much taller			4107 (5.2)
			
*Vegetarian before age 8 years*	81 416 (99.8)	190 (0.2)	
No			81 107 (99.6)
Yes			309 (0.4)

a Only three subjects reached menarche at age 7 years and six subjects at age 20 years.

b A large amount of data were missing for this variable because it was only available if the mother was also a study participant.

c Prenatal exposure to these factors.

d A large amount of data were missing for this variable because it was only available if the mother was also a study participant and the subject was a first-born child.

e The largest non-white ethnic group was Indian women (33.2%) followed by women who classed themselves as Black-Caribbean (17.3%).

**Table 2:** Effect on age at menarche from prenatal and childhood factors

		**Effect on age at menarche in months (95% CI)**	
**Risk factor**	**Unit**	**Univariate (max. *N*=81 438)**	**Multivariate (*N*=48 079)^a^**
Birthweight	Per 500 g increase	0.31 (0.19, 0.43)^***^	1.24 (1.10, 1.37)^***^
Twinning (Ref. Singleton)	Same-sex DZ twin	1.36 (−0.13, 2.86)	
	Opposite-sex DZ twin	1.75 (0.33, 3.17)^*^	
	MZ twin	2.03 (0.44, 3.62)^*^	
Maternal age at birth	Per 5-year increase	−0.08 (−0.20, 0.03)	0.36 (0.19, 0.53)^***^
Pre-eclampsia or eclampsia (Ref. Not exposed)^b^	Exposed	−1.31 (−1.93, −0.69)^***^	
Maternal smoking (Ref. Not exposed)^b^	Exposed	−1.83 (−3.23, −0.43)^*^	
Number of weeks breastfed	Per 4-week increase	0.08 (0.01, 0.15)^*^	
Ethnicity (Ref. White)	Mixed white/non-white	−1.70 (−3.59, 0.20)	−1.39 (−3.81, 1.02)
	Non-white	−3.49 (−4.84, −2.13)^***^	−7.80 (−9.90, −5.70)^***^
Weight at age 7 years	Per increase in category^c^	−5.07 (−5.23, −4.90)^***^	−4.54 (−4.75, −4.33)^***^
Height at age 7 years	Per increase in category^c^	−3.48 (−3.62, −3.34)^***^	−2.98 (−3.16, −2.80)^***^
Childhood exercise	Per 10 h increase each week	1.51 (1.18, 1.84)^***^	1.18 (0.78, 1.59)^**^
Number of siblings	Per sibling	0.96 (0.87, 1.05)^***^	1.45 (1.28, 1.61)^***^
Birth order	Per increase in birth order	0.27 (0.15, 0.40)^***^	−1.26 (−1.48, −1.03)^***^
Vegetarian before age 8 years (Ref. No)	Yes	−1.33 (−3.27, 0.62)	

Abbreviations: CI=confidence interval; DZ=dizygotic; MZ=monozygotic; Ref.=reference group.

^*^P<0.05, ^**^P<0.01, ^***^P<0.001.

Effects were estimated using ordinary least squares regression.

a The multivariate model was chosen using backward selection. Maternal smoking and number of weeks breastfed were not eligible for this model due to the large amounts of missing data for these variables.

b Prenatal exposure to these factors.

c See Table 1 for a list of the weight and height categories.

**Table 3:** Mean age at menarche (years) grouped by childhood weight, childhood height and birthweight

	**Weight at age 7 years compared with peers**			
**Variable**	**A little or much thinner**	**About the same**	**A little or much heavier**	***P*_trend_ a**
*Birthweight, g*				
<3099	13.01 (6237)^b^	12.52 (110 667)	11.99 (1928)	<0.001
3100–3399	13.15 (3349)	12.61 (79 345)	12.05 (1512)	<0.001
⩾3400	13.17 (48 645)	12.68 (15 187)	12.17 (4062)	<0.001
*P*_trend_	<0.001	<0.001	<0.001	
				
*Height at age 7 years compared with peers*				
A little or much shorter	13.37 (7367)	12.90 (7413)	12.44 (1237)	<0.001
About the same	13.08 (7111)	12.64 (29 602)	12.26 (4431)	<0.001
A little or much taller	12.82 (5793)	12.38 (11 310)	11.89 (4742)	<0.001
*P*_trend_	<0.001	<0.001	<0.001	

a *P*-values for trend are based on ordinary least-squares regression.

b Figures in brackets are numbers of participants.
